# Evolution of Low Carbon Supply Chain Research: A Systematic Bibliometric Analysis

**DOI:** 10.3390/ijerph192315541

**Published:** 2022-11-23

**Authors:** Qiang Du, Jiajie Zhou

**Affiliations:** 1 Center for Green Engineering and Sustainable Development, Chang’an University, Xi’an 710064, China; 2 School of Economics and Management, Chang’an University, Xi’an 710064, China

**Keywords:** low carbon supply chain, construction industry, literature review, hotspots, cluster analysis

## Abstract

With the increasingly serious global carbon emission problem, how to reduce carbon emissions has attracted a great deal of attention from academics and practitioners. Carbon emissions can be decreased more efficiently by coordinating the management of firms upstream and downstream in the supply chain, which has an increasingly important role in the low carbon process. Research on the low carbon supply chain (LCSC) has gradually evolved into important branches of global sustainable development. This paper aims to conduct a complete thematic analysis of 754 articles published between 2012 and 2021, identify the structural dimensions of evolution, and classify them according to systematic methodology. It provides a stage-by-stage summary of relevant research results from the past decade. At present, research in the LCSC field has resulted in a complete theoretical framework and research system and has formed the evolutionary path of method-policy-practice research. This study will help to promote further in-depth study of the LCSC and the fabrication and improvement of its theoretical system. It provides a valuable reference for researchers interested in LCSC, and points out the focus and direction of future research.

## 1. Introduction

With the significant increase in carbon emissions over the past two decades, the issue of climate change has entered the international agenda. The Copenhagen Accord and the Paris Agreement are both promoting the world to adopt quantified and effective emission reduction plans [1]. Several countries are combating climate change by incorporating “carbon neutrality” into their national strategies. A development model centered on a low carbon economy has been formed, and the concept of the low carbon transformation of supply chain systems has gradually formed [2,3]. Relevant research continues to deepen and extend this, and industrial carbon emission reduction and sustainable development have become research hotspots [4]. A supply chain is a complex system that includes many activities from raw material procurement to transportation, each of which involves emission of significant amounts of carbon. Approximately 75% of industrial sector carbon emissions are associated with supply chain operations [5,6].

A low-carbon supply chain (LCSC) is a more restrictive and competitive form of organization that takes the focus on carbon emissions even further [7,8]. In recent years, scholars have carried out research on carbon emission reduction by redesigning the supply chain network, and an optimization goal has been achieved [9]. Scholars have also conducted studies from the industry perspective. For example, Tian, et al. [10] explored the key supply chain pathways that drove CO_2_ lifecycle change in China’s manufacturing sector from 1992 to 2012. Xia, et al. [11] provided a comprehensive assessment of the key energy policy perspectives of Chinese industry about energy security, energy efficiency and carbon emissions. Single companies cannot effectively implement carbon reduction strategies because cooperation of upstream and downstream enterprises in the supply chain for carbon management is necessary to efficiently achieve carbon reduction [12,13]. Therefore, the cooperation of various parts of the supply chain can promote carbon reduction [14]. In summary, the research on LCSC is always a noteworthy topic. As mentioned above, it is especially necessary to apply the supply chain research methods of low carbon science to actual operational activities in order to improve the efficiency and energy-saving effect of supply chain operations. This has important theoretical significance and application value to conduct systematic and in-depth research on some frontier issues in the field of LCSC.

However, few studies have visualized the evolution of LCSC research and pointed out the frontier of this field based on the bibliometric visualization method. There are various measurement tools, such as BibExcel, VOSviewer, and HistCite, that can be used to analyze knowledge graphs. Such bibliometric tools allow for a systematic review of previous research, and a scientific network analysis allows for the clustering and emergence of important information [15]. CiteSpace can be used to visualize the structure of scientific knowledge, the current state of research, and the evolutionary process.

Most previous studies adopted manual review and sorting methods, and quantitative analyses of literature characteristics, evolutionary trends, and citation data were incomplete [16]. Therefore, this paper uses CiteSpace to conduct a comprehensive bibliometric study. In this study, analyzing the existing LCSC literature based on the bibliometric visualization method helps provide a clear picture of how knowledge and theories have advanced, and how research topics have evolved. This review makes three main contributions: (1) a bibliometric co-citation analysis using CiteSpace software provides a more complete and comprehensive analysis of the current state of research and hotspots in LCSC research; (2) progress of research on the LCSC in the construction industry is comprehensively and scientifically illustrated using a combination of quantitative and qualitative methods, thereby allowing for a complete understanding of the key points in this field, and (3) the study has important theoretical and practical significance for supply chain management, and for improving the level of carbon emission reductions at the industry level.

The remainder of this paper is organized as follows. Section 2 offers an explanation of the research methodology. Section 3 presents basic analysis and provides descriptive results of the literature. Section 4 determines the strength of this research field, including countries, institutions, and authors, and reveals the trends of research topics and research hotspots over time. Section 5 provides the conclusions and offers further research directions for supply chain carbon reduction.

## 2. Methodology

A systematic literature review is a comprehensive search of existing relevant literature and a critical review of qualified research to address specific subject matter research issues [17]. At the 2009 United Nations Climate Change Conference, countries reached a consensus on jointly addressing climate change and achieving low carbon development. Around 2010, the concept of LCSC attracted the attention of scholars, so we selected the research sample time range from 2011 to 2021. This not only covers the rise and peak period of LCSC research, but also better considers the development dynamics and trends of this field over the past ten years.

The Web of Science database is the world’s largest comprehensive academic information platform, covering the largest number of disciplines, making it an ideal source of data for this study. We chose the 2011–2021 period to develop the literature review collected from the Web of Science Core Collection. In the database, we first set the search conditions: TS = (“low carbon” or “carbon emission” or “low-carbon”) AND TS = (“supply chain” or “supply chains”), and 1336 documents were obtained. Two types of documents, “Articles” and “Review“, were further selected, and 1173 of them were selected. Finally, eliminating duplicate and irrelevant documents, 754 documents were used as the basic data source for systematic visualization analysis.

Bibliometric analysis is of increasing importance in the research evaluation process [18]. To get the most out of any analysis, knowing the capabilities of each tool and selecting the one that best fits your needs is essential. Histcite, Vosviewer, and CiteSpace are the three more popular literature analysis programs currently available after thorough screening and comparison. Histcite generates a citation chronology that traces the evolution of the field as a time series, enabling cluster analysis and time series analysis but not data standardization. Vosviewer can visualize research objects from multiple perspectives, construct multiple matrices, and support text mining, but it cannot de-duplicate data and display the evolution of a domain over time. CiteSpace can avoid the shortcomings of the above two software, and is a relatively complete bibliometric tool. In the whole process of building knowledge graph, each step of processing can meet the needs of different researchers.

CiteSpace software is based on co-citation analysis theory and pathfinding network algorithms [19,20], which can clearly show the trend of research objects over a certain period by quantitatively analyzing the literature in a specific research area and elucidate the complex citation network in the relationship between information in the literature (Small, 1973). Through a series of visual knowledge graphs, the dynamic mechanism of the evolution and the development frontier of a certain field are explored and analyzed [21]. In this study, CiteSpace 5.8.R3 was used to visualize and analyze literature in the LCSC field to improve the objectivity of existing relevant research advances.

CiteSpace normalizes the original co-occurrence matrices and uses them for network visualization analysis. It provides three methods for calculating the strength of connections in a network. This paper chooses Cosine to calculate the connection strength in a network. The internal relationships between documents can be more comprehensively displayed. Cluster analysis can classify a large number of keywords into different research topics based on the relative relevance of the extracted terms and put keywords into related topics, which helps to identify research topics, trends and their interconnections within the research field. In this paper, the g-index is used to extract knowledge units. The algorithm is based on increasing the scale factor k to compensate for the defect of the h-index, that is, the fact that it cannot reflect highly cited papers. Knowledge units are extracted based on the revised g-index ranking.

## 3. Descriptive Analysis

### 3.1. Definition

The concept of low carbon supply chain is based on a green supply chain and a closed-loop supply chain. It focuses the concept of “low-carbon” and integrates it into the supply chain more than the green supply chain, and explores the issue of carbon emissions in the supply chain from a new perspective. Only through cooperation of upstream and downstream enterprises in the supply chain for carbon management can we efficiently achieve carbon reduction [12,22]. Benjaafar, et al. [23] proposed development of a model that reduces the impact of carbon emissions through operational adjustments, influenced by the cooperation of companies in the same supply chain to reduce costs and emissions. For the construction supply chain, changes in demand may lead to higher carbon emissions as production becomes increasingly complex and sophisticated. As a result, supply chain analysis can be performed in terms of simplification of problems, modeling techniques, or algorithms. By introducing carbon emissions as a constraint, supply chain operations can be better studied in the context of sustainable development.

### 3.2. Quantitative Analysis of Publications

**Figure 1:**
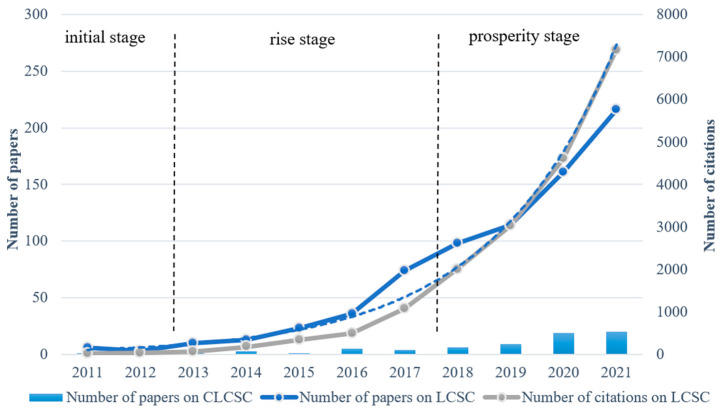
Trends in the quantity of articles and citations.

Change in the number of documents is an important indicator for measuring the progress of research in a field. The growth trend of annual publication quantity and citations is illustrated in Figure 1, which shows the trend in the quantity of articles published. Since the release of the Paris Agreement in 2015, and the fact that it has received close attention from scholars, the number of studies related to the LCSC has shown a continuous growth trend, and the number of citations has increased exponentially. An increasing growth rate was maintained year by year. In 2021, 216 papers were published, which is significantly the highest number of papers compared to previous years. The number of studies on the construction of a low-carbon supply chain (CLCSC) fluctuated within 10 articles from 2011 to 2017, and has shown a continuous upward trend from 2018 to the present, indicating that the hotness of this research continues to strengthen.

## 4. Results and Discussion

### 4.1. Co-Authorship Analysis

#### 4.1.1. Country Co-Authorship Analysis

**Figure 2:**
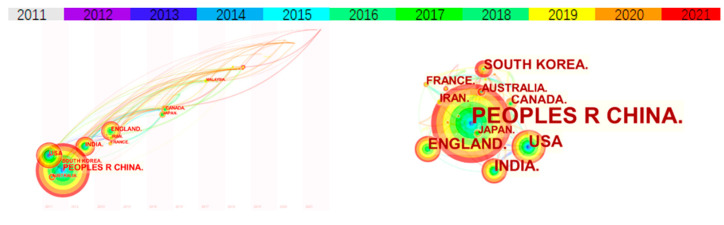
Visualization network map of the country co-authorship analysis.

The geographical locations and authors of published articles are an important indicator for measuring the academic influence of a certain field in a region. In CiteSpace, the node type “Country” is selected to analyze the country information (Figure 2).

The nodes in the figure represent different countries, and the size of the nodes is proportional to the number of studies. The time trend is reflected by the color of the connecting line, which can be seen at the top of the graph, from purple to blue, green, yellow, and orange, representing different years. The lines represent the co-occurrence relationship between different countries; the colors of the circles and lines correspond to the years, and the thickness of the rings represents centrality. As shown in Figure 2, LCSC research started in China (2011), which ranked first in the two fields, with 422 studies and a 70% share of the total number of published papers, followed by the USA and the UK. In addition, an in-depth analysis of the number of citations, the time of first publication and centrality shows that the scientific research results produced by China and the United States have great influence. China is at the core of the overall cooperation network (centrality = 0.59), connecting research cooperations with other countries. Less economically developed countries, such as Iran and India, also have well-performing publication numbers. It follows that both developed and developing countries are faced with supply chain carbon emission reduction issues.

Most developed countries that have completed the industrialization process have achieved “carbon peaking” and their carbon emissions are on a decreasing trend. They pay more attention to the closed-loop supply chain operation model with the whole life cycle of product recycling and reverse logistics. Developing countries need to construct a large number of infrastructure systems to complete the urbanization process. They must pay more attention to the front-end governance of total energy consumption control.

#### 4.1.2. Institution Co-Authorship Analysis

**Figure 3:**
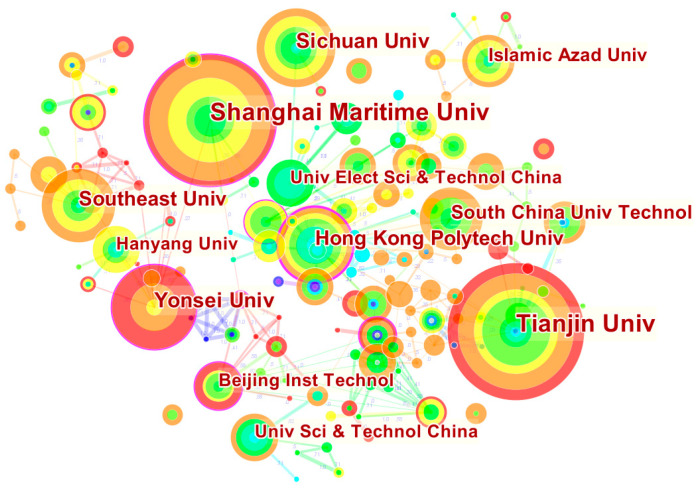
Visualization network map of the institution co-authorship analysis.

We selected the node type “Institution” in CiteSpace to analyze institution co-authorship and obtain the cooperative network map of institutions (as shown in Figure 3).

Table 1 shows the top five institutions in terms of the total number of publications. In Figure 3, there are 256 nodes and 315 connections, and the cooperative network density is 0.0097. The nodes of Tianjin University and Shanghai Maritime University have more external connections. Most institutions are independent in the network, indicating that collaboration among these research institutions is relatively fragmented. Notably, Chinese institutions account for 80% of the top five institutions, with a wealth of research. Hong Kong Polytechnic University has the highest centrality (0.25), indicating that this node is the key node in the whole network.

#### 4.1.3. Author Co-Authorship Analysis

**Figure 4:**
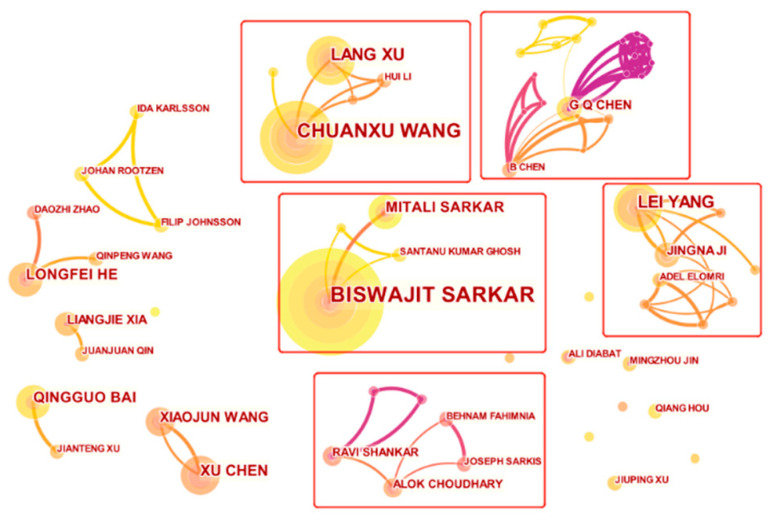
Visualization network map of the author co-authorship analysis.

The top eight most influential authors are sorted in Table 2 by the number of citations they gained. After eliminating isolated nodes, selecting the k = 25 threshold, and setting the unit of analysis, the collaboration among researchers was evaluated, as demonstrated in Figure 4.

As shown in Figure 4, five representative incipient collaborative groups have formed in the LCSC field, and BISWAJIT SARKAR is the most influential author. Small internal networks centered on CHUANXU WANG, G Q CHEN, and LEI YANG were formed. Most other points are scattered and have not formed a great cooperation network. To summarize, some existing research focuses on collaboration in small groups, and some authors publish their articles independently.

The institutions and countries of the authors mentioned in the table are basically consistent with the majority of research institutions mentioned in the previous section, indicating the formation of a group of leading scholars who are representative.

### 4.2. Research Frontier Analysis

#### 4.2.1. Document Co-Citation Analysis

The co-citation relationship of documents changes over time and can be used to explore the development and evolution of a discipline. High-frequency co-citation documents show similar research ideas and or a similar focus in the field, and they are a powerful basic reference for subsequent researchers [24]. We selected the most representative papers, the top five co-citation papers in the Table 3, from all studies for further analysis.

Benjaafar, Li and Daskin [23] explained the concept of the carbon footprint in supply chain management, incorporating carbon emission issues into operational decisions in procurement, production, and inventory management. This study has the highest number of citations, and it is the basis for the later decision-making problems in this field. Ji, et al. [25] used a Stackelberg game model to study the emission reduction behavior of chain enterprises in the case of retail channels and dual channels to provide an effective reference for subsequent policy development and implementation. Wang, et al. [26] established a binary supply chain game model for manufacturers and retailers and studied the carbon emission reduction problems in the two situations of retailer dominance and power balance. Du, et al. [27] studied the impact of emission cap and trade mechanisms on the supply chain of emission permit suppliers and emission-related companies, and showed that the operation of the LCSC by different carbon regulatory policies is a research hotspot in this field. Xu, et al. [28] proposed that both wholesale prices and cost-sharing contracts can coordinate supply chain issues under cap-and-trade regulation. After these detailed analyses were obtained, five articles clarified the development trend from different directions, from the micro to the macro, and from the low carbon behavior of supply chain participants to optimization problems under policy constraints.

**Figure 5:**
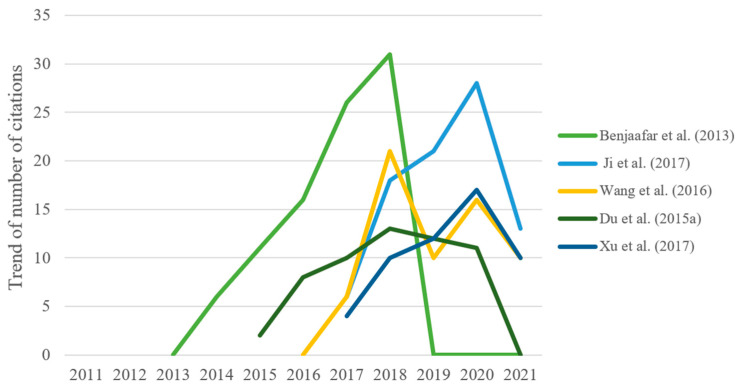
Citation history analysis of the top five co-citation documents.

Figure 5 shows the citation history trend of the last ten years in the five articles mentioned above. Most of them have higher citation counts from 2016 onward, basically showing a trend of rapid growth to a peak and then decline. Notably, No. 3 [26] was published in 2016 and reached two peaks in 2018 and 2020, which indicates that in regard to emission reduction, corporate low carbon transition has been the focus of attention in recent years.

In addition, it can be seen from the high co-citation references that the research basis mainly includes the following: (1) relevant research fields include green supply chain management and sustainable supply chain management, as well as low carbon practice policies; (2) identification and evaluation of the carbon footprint of the supply chain, and (3) optimization research methods in operations research.

#### 4.2.2. Keyword Co-Occurrence

Keywords are highly generalized and concentrated descriptions of the core content of the literature, and they can intuitively reflect the research hotspots and research trends in a field and condense the changing trends of research theories, research perspectives, and research methods. The relationship between different keywords in the same document can also reveal the internal relationship between disciplines to a certain extent. In this section, keyword and document co-citations are used as node types to profile and summarize the research trends and hotspots in the LCSC field.

Keyword co-occurrence analyzes the literature information from a microscopic perspective, focusing on the research object and research focus of the literature. In this paper, the co-word analysis of keywords is used to determine the research hotspots of LCSC applications. In CiteSpace, the node “keywords” is chosen, the authors’ original keywords are extracted, the year slice is set to 1 year, the connection rule is a path-finding network, the threshold of the top 50 is selected, and the graph is pruned and simplified to construct a keyword co-occurrence network. Similar keywords, for example, “emission” and “CO_2_”, are merged into “carbon emission”, and “LCA” is merged into “life cycle assessment”. The larger the nodes in the graph and the thicker the connecting lines are, the more in-depth the research on the keyword, and the more closely linked the relevant research.

**Figure 6:**
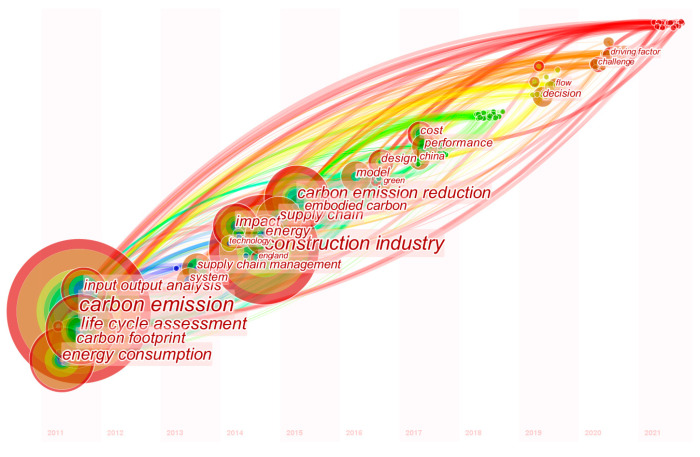
A visualization of LCSC keyword co-occurrence network.

In the keyword co-occurrence map, 412 nodes and 2484 connections are formed. Figure 6 shows the keywords co-occurring along the timeline to reveal the emergent trends in recent years. As shown by the three stages of development, most of the high-frequency keywords focus on the following four aspects. (1) The first is about supply chain management: supply chain management (frequency = 148), supply chain coordination (frequency = 143), optimization (frequency = 102). (2) The second is about the research methodology: model (frequency = 136), carbon footprint (frequency = 68), game theory (frequency = 76), network design (frequency = 30). (3) The third relates to supply chain stakeholders: manufacturer (frequency = 31), consumer environmental awareness (frequency = 27), retailer (frequency = 18), consumer (frequency = 18), remanufacturing (frequency = 16). (4) The fourth concerns low carbon policy: cap and trade (frequency= 83), carbon tax (frequency = 56).

The keyword map is displayed based on the “Timezone” (Figure 6), and the corresponding high-frequency words are extracted (Table 4). The research process was divided into three stages based on the keyword information and how it was presented and the quantitative analysis of the publications in Section 3.2. The initial stage was from 2011 to 2012. The relevant research was mainly focused on carbon emissions from the four nodes of the carbon footprint, input-output analysis, life cycle assessment, and energy consumption. Related research focused on how to measure supply chain carbon emissions. The rising stage was from 2013 to 2017. The number of key nodes in this stage increased significantly, and the research content was continuously extended and expanded, gradually forming a clear research background and branches. The focus was mainly on issues related to supply chain management, influencing factors and technological progress from a macro perspective. The prosperity stage was from 2013 to 2017, and related research paid more attention to the challenges brought by the supply chain operation cost, performance and the complex and the changeable external environment.

There are currently three types of carbon emission policies, mandatory emission policy (also known as cap emission policy or carbon cap policy), carbon tax policy and carbon trading policy (also known as cap-and-trade policy). At present, governments of various countries have formulated and implemented various types of low carbon policies. Most of these target the general industrial sector, with few low-carbon policies for the construction sector.

The results show that scholars started with methods to conduct research on the LCSC and carry out relevant research on the low-carbon operation of the supply chain based on supply chain management, coordination, integration and optimization. From this it can be seen that in the past ten years a research system has been formed, and the research scope and research fields have become increasingly extensive.

### 4.3. Evolutionary Trends Analysis

#### 4.3.1. Clustering Analysis

We used the Log-Likelihood Ratio (LLR) algorithm to select the best clustering labels based on uniqueness and coverage and to generate high cluster quality, high intraclass similarity, and low interclass similarity. These indicators objectively reflect the research hotspots in the research field. The size of a node represents the degree of a hotspot (the larger the node, the higher the popularity). The relationship between different keywords is shown by the node connection. The thicker the connections, the stronger the correlation between them. Regarding the generated clustering index, the clustering module value (Modularity, Q value), it is generally believed that Q > 0.3 means that the clustering structure is significant. Regarding the clustering average contour value (Silhouette, S value), it is generally believed that Silhouette > 0.5 is reasonable clustering, and Silhouette > 0.7 means the clustering is convincing. Through Table 5, it can be seen that, the modularity was greater than 0.3, and the Silhouette was greater than 0.75, indicating that the formed clusters are highly credible and objectively show the subject of related research.

**Figure 7:**
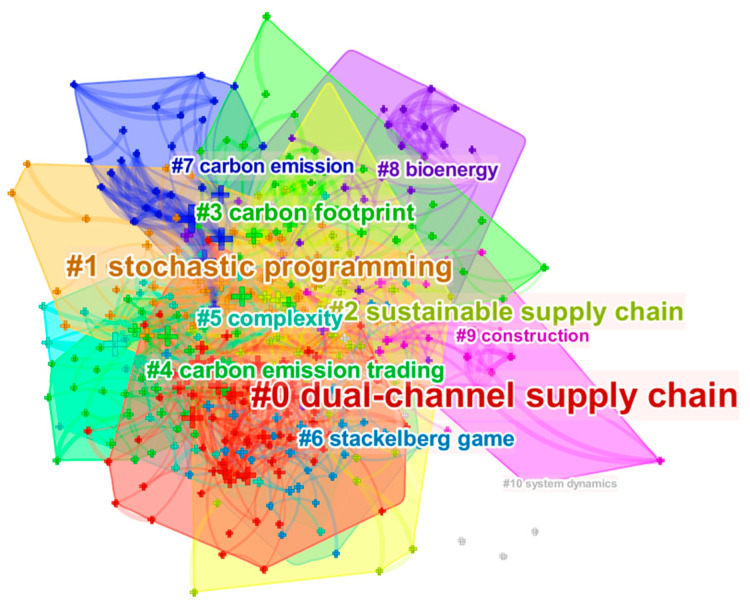
LCSC keyword clustering.

(1) As shown in Figure 7, a total of 11 clusters were generated, of which cluster #0, the dual-channel supply chain, was the cluster with the largest capacity, containing 82 papers. Derivatives belong to the same LCSC as cluster #2 sustainable supply chain; Cluster #6 Stackelberg game has the most recent average citation year (2018), and cluster #1 stochastic programming, cluster #10 system dynamics and cluster #3 carbon footprint are research methods for the study [29]. The time of appearance indicates the changing trends and evolutionary characteristics of the research methods. From the original mathematical planning problem, research has gradually moved to the use of operational research methods and game theory to solve optimization problems in various complex LCSC scenarios. In summary, this work provides a clear view of the evolutionary development path of the field.

Overall, the research themes of the past ten years have been more concentrated, and the research directions tended to be diversified and cover a wide range of areas, with crossover phenomena.

Cluster #0 dual-channel supply chain, with an average publication year of 2017, was the largest cluster, with 82 papers. It includes cap-and-trade, low-carbon preference and other keywords. In this cluster, researchers focused on carbon reduction in different supply chain forms and policy-bound preferences for low-carbon behavior [30].

Cluster #1 stochastic programming had an average publication year of 2016 and includes 57 articles. It contains carbon policies, the closed-loop supply chain, uncertainty and other keywords. In this cluster, researchers focused on modeling and optimizing supply chains under multiple scenarios through a mathematical planning approach [31,32]. These studies further illustrate that stochastic programming is the main research method in the emerging stage (2013–2016).

Cluster #2 sustainable supply chain had an average publication year of 2015. It includes supplier evaluation, low carbon, performance evaluation and other keywords. Different from dual-channel supply chains, in this cluster, researchers focused on the issue of balance with environmental sustainability under multiple factor combinations [33,34,35].

Cluster #3 carbon footprint had an average publication year of 2015. It includes international trade, input–output analysis and other keywords. In this cluster, researchers focused on the application of carbon accounting methods [36].

Cluster #4 carbon emission trading had an average publication year of 2017. It includes deteriorating items, inventory, imperfect quality and other keywords. In this cluster, researchers focused on the impact of relevant low carbon policies on supply chain management decisions [37].

Cluster #5 complexity had an average publication year of 2016. It includes economic order quantity, carbon regulations, financing and other keywords. The main focus was on optimizing costs, carbon regulation, and other complex issues in supply chain operation [38].

Cluster #6 Stackelberg game had an average publication year of 2018. It includes differential game, games, green products and other keywords. In this cluster, researchers focused on the application of multiple game frameworks to the low carbon operation of supply chain [39,40].

Cluster #7 carbon emission had an average publication year of 2012, which is the earliest, and it had the highest Silhouette. It includes goal programming, information asymmetry, supply chain management and other keywords. In this cluster, the focus was on the impact of carbon emission on supply chain management (including supply chain network design, inventory management, information resource matching, etc.) [41].

Cluster #8 bioenergy had an average publication year of 2015. It includes technology, renewable energy and other keywords. Researchers focused on the challenges posed by new energy and new technologies to supply chain decarbonization [42].

Cluster #9 construction had an average publication year of 2016. This cluster contains embodied carbon, building materials and other keywords. In this cluster, researchers focused on low carbon issues in the construction supply chain [43]. In the next subsection, the relevant studies are discussed in depth.

Cluster #10 system dynamics had an average publication year of 2015, and it covers theoretical analysis, incentive policy, partnership and other keywords. In this cluster, researchers focused on the integration of low-carbon policy and low-carbon practice [44].

From further analysis in Table 6, it can be concluded that research on LCSC was mainly based on quantitative model optimization and qualitative research based on survey analysis. Among these, game theory and its correlations were used to study the development of optimal reduction strategies, using mixed integer programming, stochastic programming and other methods to solve LCSC location and path optimization problems. Some scholars also used different algorithms to design low-carbon networks.

(2) Buildings account for 40% of the world’s total energy use, which has a major impact on greenhouse gas emissions and global climate change [45]. As one of the main industries of the national economy, the construction industry has enormous carbon emissions. In addition, it has had a significant impact on the environment and consumes vast amounts of resources in the process of development [46,47]. The COVID-19 outbreak has had a severe impact on the global construction industry, with construction activity showing a 10–25% reduction compared to 2019. Global carbon emissions from the construction and operation of the building sector reached 14 billion tons in 2020, rising to a historical high of 38% of total global carbon emissions. Therefore, low carbon development in the construction industry should be taken seriously [48].

Construction supply chain (CSC) is a complex system consisting of multiple subjects, such as customers, contractors, designers, subcontractors and suppliers, who are interconnected through the product and material flow, capital flow and information flow [49]. Scholars have outlined the development of the supply chain in the construction industry [50,51]. Aloini, et al. [52] used a literature review approach to classify and analyze 140 papers from a risk management perspective in the construction industry. Shi, et al. [53] provided a systematic review of mobile internet-based CSC management, establishing a comprehensive framework that includes material flow and supply management, real-time information sharing and communication, coordination and integration in the CSC, technical support for mobile Internet, and related security issues. Wang, et al. [54] focused on prefabricated supply chain management and proposed a new classification method in terms of literature characteristics, research methods and future trends. However, there is a lack of a systematic reviews on LCSC issues in the construction industry (Dallasega et al., 2018).

Notably, construction is the only industry that appears in all the formed clusters. This result further indicates that a research base has been developed for supply chain carbon reduction from an industry perspective. Due to the large carbon emissions in the construction industry, the role of indirect, complex and interconnected supply chains in carbon emission reduction in the construction industry must be considered [55]. Therefore, we selected 69 articles on the CLCSC from the 754 studies for further study in an attempt to clarify their evolutionary paths. Chen et al. (2011) proposed building construction, decoration, outdoor facility construction, transportation, operation, waste treatment, property management, and building demolition and disposal, as the nine stages of carbon emission accounting. Their paper marks the beginning of research on carbon reduction in the construction industry.

A total of eight clusters were generated, but it is difficult to accurately identify deeper information only from the labels of the clusters. Therefore, it is necessary to analyze each cluster by combining the main keywords and key documents contained in the clusters. Regarding the specific research content in the text, based on the clustering capacity, we mainly list the first five clusters in Table 7.

**Figure 8:**
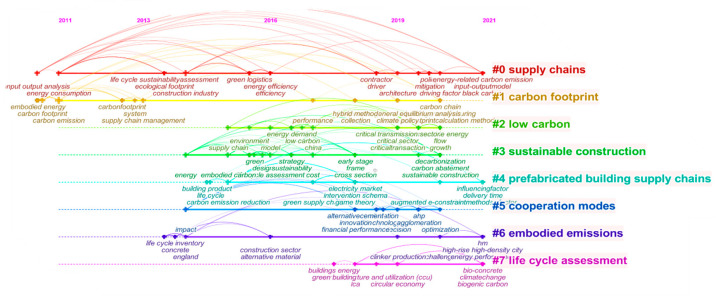
Clustering of the CLCSC.

In Figure 8, a node represents a keyword, and the size of the node is proportional to the frequency of the keyword. Nodes corresponding to the frequency of occurrence of the keyword are highlighted with a cross. Keywords from the same year are connected by lines of the same color. Different colored lines are used to link keywords that appear in different years. A cluster represents a research area, and the smaller the number of clusters (closer to 0), the more keywords the cluster contains.

Cluster #0 supply chains includes 33 papers with an average publication year of 2016. These papers mainly discuss the framework formation of low-carbon building supply chains [56]. Some scholars have proposed that the optimization of building materials is the focus of supply chain emission reduction and requires attention [65].

Cluster #1 (carbon footprint) includes 24 articles with an average publication year of 2014, and it is the same as LCSC cluster #3. It emphasizes the timely collection and calculation of the carbon footprint of all types of buildings, allowing for a reasonable quantification of carbon emissions throughout the supply chain [58,66]. Sun [57] proposed the use of renewable energy, improved construction technology, and an integrated supply chain to realize carbon emission reduction across the supply chain and establish an effective energy-saving and emission reduction mechanism. This cluster of the literature tends to be more focused on material recycling or reuse or product life cycle assessment [67].

Cluster #2 low carbon includes 24 articles with an average publication year of 2018. Ranging from project management [59], technological progress, and policy regulation [68] and other different influencing factors has been conducted in-depth research on the development of low carbon buildings.

Cluster #3 sustainable construction contains 23 articles with an average publication year of 2017. Interpreted in conjunction with LCSC cluster #2, it is easy to see that low carbon and sustainability are two concepts that cannot be separated. D’Amico and Pomponi [69] found a method that can support builders in considering implicit carbon factors early in the design process by incorporating low carbon concepts in the design phase. By configuring the supply chain to use resources with lower emissions, carbon emissions in the process can be effectively controlled [63].

In Cluster #4 prefabricated building supply chains, 20 papers were cited. The average publication year was 2017. Prefabricated buildings provide an important opportunity for structural carbon emission reduction in the industry and are an important method of carbon reduction in the construction industry. As an effective solution to carbon emissions [70], the construction method can shorten the construction period and improve the utilization rate of materials [71,72], achieving carbon reduction in the construction process. This establishes the direction for the development of low carbon buildings [73].

Carbon emission reduction at the industry level is imminent. Some of these reviews have analyzed the development of supply chain management introduction in the construction industry [52,53,54], while others have focused on construction supply chain modeling and participant relationships [74,75,76].

#### 4.3.2. Burst Analysis

The burst detection function provided by CiteSpace can discover the decline or rise of a certain keyword based on the distribution of burst keywords corresponding to topics [77]. If a citation or keyword has received special attention from scholars over time and the citation frequency increases rapidly, the citation or keyword is a research frontier in the field. The citation nodes that emerge are shown in red, red indicates the duration of the burst, and the intensity indicates the rate of increase in citation or keyword frequency. Table 8 and Table 9 list the top five references with the most prominent citations in the LCSC and CLCSC fields, respectively.

Benjaafar, Li and Daskin [23] incorporated carbon emissions into operational decisions in procurement, production and inventory management using a classic operational management model, and they optimized the supply chain while considering cost and carbon footprint decisions. Their paper is also the most cited and influential article in this field. In other words, follow-up research is based on this article. Chaabane, Ramudhin and Paquet [78] added lifecycle assessment principles based on considering the cost-effectiveness of each node in the construction supply chain and designed a sustainable supply chain design framework to help decision makers achieve sustainable development targets [71]. Hua, et al. [42] applied a carbon emission trading mechanism to inventory management, balancing the optimal order quantity and carbon footprint. Du, Zhu, Liang and Ma [79] conducted an in-depth analysis of the relationship between environmental policy constraints and manufacturers’ profits. Liu, Anderson and Cruz [80] focused on the impact of competition and consumers’ environmental awareness on key supply chain players.

Chau, et al. [84] considered the impact of different factors (building materials, construction methods, and transportation methods) on building carbon emissions. This document is the earliest of the 57 articles to have an impact (2015). Atmaca and Atmaca [83] compared the carbon emissions of buildings in rural areas and buildings in urban areas throughout their lifecycle and found that buildings in urban areas have higher carbon emissions than rural areas carbon emissions, per square meter, during the operation phase. Additionally, they considered the potential impact of the construction process on the environment to reduce energy, materials and land use. Bing, et al. [82] considered the emissions trading constraints, and the network was optimized using integer programming methods. Chaabane, Ramudhin and Paquet [78] redesigned the reverse supply chain from a global perspective through a case study to minimize the costs of the global chain under an emissions trading scheme (ETS) (collection, transportation and processing, and carbon trading costs). Azari and Kim [81] proposed a comprehensive and quantifiable integration assessment framework adapted to the context of green buildings, and their paper is the most influential study.

Keywords are a set of technical terms that reflect the core content of a research article [85]. In order of intensity, in the past ten years, the LCSC field has entered a period of high activity, and many high-intensity words have emerged in Table 10. “Carbon footprint” and “inventory” are the two with the highest intensity. Emerging words indicate that the consumption-based and production-based perspectives complement each other. Carbon emission measurement (carbon footprint, LCA, etc.) and inventory management were early research hotspots in the LCSC field. Game theory is a combination of operations research and mathematics combined with quantitative research tools, and it was a hotspot in the 2017–2018 period. “Cost” and “decision” are prominent words with higher intensity in the CLCSC field, which further illustrates the depth of this field. Research arises from the cost control problem under low carbon constraints.

In the last decade, the LCSC has also gradually evolved from management science to a multidisciplinary blend of fields with three key foundational knowledge bases: the optimization of carbon emission measurement methods and the development and methodological framework of multiagent coordination under carbon policy regulation.

## 5. Summary and Conclusions

A systematic visualization bibliometric method was used to visualize 754 LCSC articles from 2012 to 2021 in the WOS core database, and to conduct an in-depth research of 69 CLCSC articles. The review summarizes the research progress in the period under study. Meanwhile, the evolutionary structure and research frontiers are presented as well, providing a useful understanding of this emerging research and furnishing valuable information for future directions. The conclusions are as follow:

(1) Regarding the main drivers of research, scholars in China and elsewhere have carried out in-depth research on the LCSC, and an accumulation of research has been achieved. From the number of published papers, citations and published journals, it can be seen that the low carbon trend is an important branch of supply chain research worthy of continued study, and the upgrading of the CLCSC is also the focus of global scholars. It can be seen from the main countries and core scientific research institutions that published papers that China, the UK and the USA have greater scientific research influence, and an increasing number of high-quality journals are also focusing on the low carbon development of the supply chain.
(2) A comprehensive literature review shows that the LCSC is a hot topic. The analysis of emerging keywords shows that new research hotspots or frontiers are mainly focused on the accounting methods for carbon emissions in supply chains, optimization problems in LCSC management, and cooperative relationships between stakeholders from a systemic perspective. This information can better guide the direction of research on the LCSC and obtain more valuable research results. From the analysis of the construction industry, it can be seen that it is very necessary and important to conduct LCSC research at the industry level, as such research can provide new ideas for emission reduction in the transportation industry and power industry with large carbon emissions.
(3) According to the co-citation analysis, there are eleven major research directions in the LCSC field, including construction. It is shown that the measurement and evaluation of carbon emissions in the supply chain at the industry level are very important to formulate scientific and effective carbon emission reduction policies. LCSC research is marked by dynamic changes in terms of the evolution of keywords under the influence of the external environment, such as particularly information technology and COVID-19. The boundaries of LCSC research are still expanding.

In summary, existing research mainly focuses on three aspects: supply chain carbon emission measurement methods; supply chain emission reduction optimization under environmental regulation policies, and supply chain management and operations [86], such as procurement, costing, distribution, network design and supply chain coordination.

In addition, Throughout the increasingly complicated process of low carbon supply chain management, it is necessary to employ multidisciplinary principles to solve practical issues, which can be studied in the following ways.

(1) Efforts should be made to find a supply chain-based industry emission reduction framework that balances economic and environmental benefits to provide a reference for supply chain sustainability related research. It is important to conduct more research on carbon reduction in the construction industry.
(2) Research on countermeasures for specific problems should be strengthened to explore the dual effects of emission reduction and increasing income in the construction industry [87,88]. It is necessary to consider incorporating external factors such as government subsidies and support from financial instruments, and internal factors such as stakeholders’ preferences for low carbon attitudes into the study of CLCSC, which are used to achieve emission reduction targets.
(3) International variation in carbon emission policy complicates related studies. Most of the research now focuses on single, low-carbon policies, whereas future research could delve into supply chain management and network design under the complementary use of carbon taxes and carbon trading, combining a heuristics approach to explore multi-objective optimization under carbon regulation policies.

Data can be updated regularly, and related research can be carried out to further improve the knowledge map provided by this research.

## Figures and Tables

**Table 1:** Productive organizations and countries (top five).

Institution	Country	Counts	Centrality	Year
Tianjin University	China	26	0.08	2014
Shanghai Maritime University	China	25	0.13	2017
Yonsei University	South Korea	26	0.18	2019
Sichuan University	China	15	0.01	2015
The Hong Kong Polytechnic University	China	14	0.25	2011

**Table 2:** Top 8 active authors.

No	Count	Year	Authors	Institution
1	22	2015	BISWAJIT SARKAR	Yonsei University
2	15	2017	CHUANXU WANG	Shanghai Maritime University
3	10	2014	LEI YANG	South China University of Technology
4	10	2017	LANG XU	Shanghai Maritime University
5	8	2016	XU CHEN	University of Electronic Science and Technology of China
6	7	2014	LONGFEI HE	Tianjin University
7	7	2016	MITALI SARKAR	Department of Industrial Engineering, Seoul National University
8	7	2018	QINGGUO BAI	Qufu Normal University

**Table 3:** Top five co-citation documents.

	Author	Year	Title	Source	Count	Centrality	Citations
1	Saif Benjaafar	2013	Carbon footprint and the management of supply chains: insights from simple models	IEEE Transactions on Automation Science and Engineering	90	0.05	1190
2	Jingna Ji	2017	Carbon emission reduction decisions in the retail-/dual-channel supply chain with consumers’ preference	Journal of Cleaner Production	86	0.03	239
3	Qinpeng Wang	2016	Contracting emission reduction for supply chains considering market low-carbon preference	Journal of Cleaner Production	63	0.03	160
4	Shaofu Du	2015	Game-theoretic analysis for an emission-dependent supply chain in a ‘cap-and-trade’ system	Annals of Operations Research	58	0.03	229
5	Xiaoping Xu	2017	Supply chain coordination with green technology under cap-and-trade regulation	International Journal of Production Economics	53	0.04	261

**Table 4:** Visualization of the keyword co-occurrence network.

Keyword	Citation	Centrality	Year
supply chain management	147	0.09	2011
model	136	0.03	2012
supply chain coordination	132	0.02	2015
policy	103	0.03	2015
optimization	102	0.04	2015

Note: The core search terms carbon emission (228 times), supply chain (148 times), carbon emission reduction (128 times) has been removed.

**Table 5:** Cluster information.

Clustering Characteristics		Value
Cluster quality	Modularity	0.5244
	Silhouette	0.7588
Cluster quantity		11

**Table 6:** Main clusters and their detailed information.

		Cluster Label (LLR)	Main Keywords	Capacity	Silhouette	Year
Methods	#1	stochastic programming	carbon policies; closed-loop supply chain; uncertainty	57	0.677	2016
	#3	carbon footprint	international trade; footprint; input-output analysis	36	0.744	2015
	#6	stackelberg game	differential game; games; green products	31	0.795	2018
	#10	system dynamics	theoretical analysis; incentive policy; partnership	9	0.92	2015
Object	#0	dual-channel supply chain	cap-and-trade; low-carbon preference	82	0.6	2017
	#2	sustainable supply chain	supplier evaluation; low carbon; performance evaluation	47	0.788	2015
	#8	bioenergy	technology; renewable energy	27	0.852	2015
	#9	construction	embodied carbon; building materials buildings	20	0.903	2016
Policy	#4	carbon emission trading	deteriorating items; inventory; imperfect quality	35	0.813	2017

**Table 7:** Top-ranked clusters and the main keywords within the clusters.

No	Capacity	Cluster Label (LLR)	Silhouette	Keywords	Representative Literature	Year
#0	33	supply chains	0.831	construction industry; developers; input output analysis	Chen, et al. [56]; Giesekam, Barrett, Taylor and Owen [47]	2016
#1	24	carbon footprint	0.928	national climate policy; building manufacturing	Sun [57]; Hong, et al. [58]	2014
#2	24	low carbon	0.849	simulation; flow; greening global value chains	Papachristos, et al. [59]; Chen, et al. [60]	2018
#3	23	sustainable construction	0.842	carbon abatement; decarbonization	Seo, et al. [61]; He, et al. [62]	2017
#4	20	prefabricated building supply chains	0.837	intervention schemas; delivery time; governmental regulation; structural equation modeling	Waltho, et al. [63], He, et al. [64]	2017

**Table 8:** Top 5 LCSC papers ranked by burst detection.

References	Year	Strength	Begin	End	2011–2021
Benjaafar, Li and Daskin [23]	2013	23.07	2014	2018	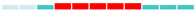
Chaabane, et al. [78]	2012	14.52	2013	2017	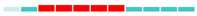
Hua, et al. [42]	2011	12	2014	2016	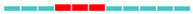
Du, et al. [79]	2013	10.08	2014	2018	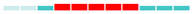
Liu, et al. [80]	2012	10.03	2015	2017	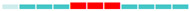

**Table 9:** Top 5 CLCSC papers ranked by burst detection.

References	Year	Strength	Begin	End	2011–2021
Azari and Kim [81]	2016	1.04	2018	2019	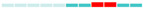
Bing, et al. [82]	2015	1.02	2016	2017	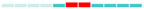
Chaabane, et al. [78]	2012	1.02	2016	2017	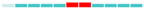
Atmaca and Atmaca [83]	2015	1.02	2016	2017	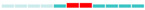
Chau, et al. [84]	2012	0.91	2015	2017	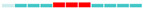

**Table 10:** Top 10 keywords ranked by burst detection.

Field	Keywords	Year	Strength	Begin	End	2011–2021
LCSC	carbon footprint	2011	7.55	2011	2016	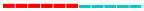
	inventory	2011	3	2015	2016	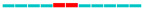
	game theory	2011	2.69	2017	2018	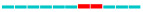
	green logistics	2011	2.6	2016	2017	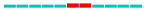
	design	2011	2.4	2013	2016	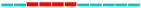
	carbon emission	2011	2.3	2013	2014	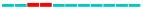
	facility location	2011	2.24	2017	2018	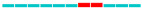
	choice	2011	2.2	2015	2017	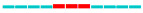
	distribution system	2011	2.18	2015	2016	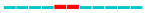
	stochastic demand	2011	2.08	2016	2017	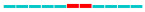
CLCSC	cost	2011	1.19	2017	2019	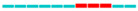
	decision	2011	1.15	2019	2021	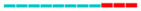
	embodied carbon	2011	1.15	2015	2016	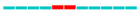
	input output analysis	2011	1.11	2011	2014	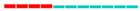
	carbon emission reduction	2011	1.03	2015	2016	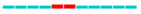
	green supply chain	2011	0.98	2017	2018	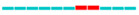
	carbon footprint	2011	0.98	2011	2013	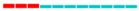
	environment	2011	0.95	2016	2017	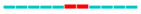
	life cycle inventory	2011	0.94	2014	2016	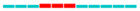
	attitude	2011	0.94	2014	2016	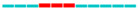

## Data Availability

Not applicable.
